# The Effects of Magnesium Sulphate on Integrated Pulmonary Index Scores and Propofol Consumption During Endobronchial Ultrasonography: A Retrospective Study

**DOI:** 10.7759/cureus.44880

**Published:** 2023-09-08

**Authors:** Şenay C Adıgüzel, Dilan Akyurt, Nevra Gullu Arslan, Mustafa Süren

**Affiliations:** 1 Anesthesiology and Reanimation, Samsun University, Samsun, TUR; 2 Anesthesiology and Reanimation, Samsun University School of Medicine, Samsun Training and Research Hospital, Samsun, TUR; 3 Pulmonary Medicine, Samsun University School of Medicine, Samsun Training and Research Hospital, Samsun, TUR

**Keywords:** propofol-based sedation, integrated pulmonary index, ebus-tbna, sedoanalgesia, magnesium sulphate

## Abstract

Aim

Our aim in this study was to investigate the effect of inhaled and intravenous (iv) magnesium (Mg) use on Integrated Pulmonary Index (IPI) score and propofol consumption in patients undergoing endobronchial ultrasonography (EBUS) procedure under sedoanalgesia.

Materials and methods

After obtaining the approval of the local ethics committee, the files of 96 patients aged 18-75 who underwent EBUS were reviewed retrospectively. Patients using Mg were classified as the M group, and patients not using Mg were classified as the control (C) group. IPI values, amount of propofol consumed, and intubation scores of group M and group C were evaluated.

Results

When the intubation score values ​​at the time of the bronchoscope passing through the vocal cords (assessment of vocal cord movement, cough reflex, and leg movement) during the EBUS procedure were compared, the intubation conditions were found to be significantly better in the M group than in the C group (p<0.05). Group M had less cough reflex than group C (p<0.05). IPI scores were significantly higher in the M group than in the C group at the 10th and 15th minutes (p<0.05). Total propofol consumption was found to be significantly lower in the M group (254.61±82.80 mg) than in the C group (321.25±90.04 mg) (p<0.05).

Conclusion

According to our results, the use of intravenous and inhaler Mg in addition to propofol sedation during the EBUS procedure may improve the respiratory parameters and can also significantly reduce the propofol dose.

## Introduction

Endobronchial ultrasonography-guided transbronchial needle aspiration (EBUS-TBNA) is a technique that provides imaging and sampling of structures adjacent to the bronchial wall while bronchoscopy is being performed and is an effective method for diagnosing and staging lung diseases [1-3]. Sedoanalgesia is essential for patient comfort and procedure quality during endobronchial ultrasound (EBUS). It is an essential component of the procedure because it improves patient comfort and may increase diagnostic accessibility [2]. During the procedure, coughing, feeling of choking, and pain may serve as a lifelong reminder of a negative experience. Sedation during the procedure reduces these complaints; however, it can also cause additional issues [4]. Undesired events can still occur despite the best efforts to prevent them. Anesthesia management for EBUS patients is more difficult than for patients undergoing other external anesthesia procedures. The risk of complications increases when treating patients with severe airway issues. Respiratory parameters must be closely monitored when adjusting the level of sedation. The type and quantity of anesthetic agent used during the EBUS procedure are critical. Propofol is the agent of choice for short and routine procedures because it provides a good level of sedation, hemodynamic stability, amnesia, and rapid recovery in these patients [4]. In some facilities, general anesthesia regimes (including muscle relaxants) may be used.

Magnesium is an essential mineral that is found in high concentrations throughout the human body, ranking as the fourth most abundant extracellular cation and the second most abundant intracellular cation, respectively [5,6,7]. Clinically, magnesium is recognized for its analgesic, sedative, neuroprotective, cardiac rhythm regulator, bronchodilator, and anti-inflammatory properties. As a calcium antagonist, it exerts a bronchodilator effect in asthma by inhibiting the contraction of smooth muscle cells [5]. It reduces adverse effects by potentiating some anesthetic drugs and lowering dosage requirements [7]. Magnesium blocks pain-sensing nociceptive receptors by acting as an N-methyl-D-aspartate (NMDA) receptor antagonist and by opposing calcium. Consequently, it has analgesic properties [7]. It also inhibits neurotransmitter release by inhibiting NMDA. With its sedative, anticonvulsant, and neuroprotective effects, it is particularly useful for patients with preeclampsia and pheochromocytoma. In anesthesia, it is used for hypnotic and analgesic effects by reducing the need for sevoflurane, desflurane, propofol, and opioids. Due to its neuroprotective features, it is utilized in various medical fields, including neurology, pediatrics, gynecology, and neuro-anesthesia [8]

Anesthesiologists' working areas are expanding beyond the operating room and expanding constantly. It is necessary to establish fundamental standards by considering patient safety outside of the operating room. The American Society of Anesthesiology (ASA) and the Turkish Society of Anesthesiology and Reanimation (TARD) established standards of anesthesia practice in non-operating room settings [9]. In EBUS procedures, which are one of the non-operating room anesthesia (NORA) applications, the evaluation of respiratory parameters must be performed by the standards, particularly due to the respiratory pathology of the patients and the region to be operated on. The Integrated Pulmonary Index (IPI) algorithm provides a single value that represents respiratory parameters that include end-tidal carbon dioxide concentration (EtCO2), respiratory rate (SS), heart rate (HR), and peripheral oxygen saturation (SpO2). IPI enables the clinician to assess the patient's respiratory status and determine the need for intervention quickly [1,10,11]. In addition, the Food and Drug Administration of the United States (FDA) states that IPI monitors can be used to monitor respiratory parameters in sedated patients [12,13].

The purpose of this study was to evaluate the impact of inhaled and intravenous (IV) magnesium on IPI score and propofol consumption in patients undergoing EBUS under sedoanalgesia.

## Materials and methods

The study was approved by the local ethics committee (SUKAEK-2023 2/20). The medical records of patients who underwent an EBUS procedure between January 2022 and October 2022 were analyzed retrospectively. Data were obtained by accessing patient files from the hospital's archive records and by using our hospital's information processing system (Fonet v4.21.1.1).

The study included patients with ASA classifications II-III aged 18 to 75 years. Patients with morbid obesity and severe respiratory failure were excluded from the study. Patients with advanced respiratory failure were defined as those having an oxygen saturation of <90% and a dyspnea score of 4 despite the administration of 4 liters/minute (l/min) oxygen (O2) via nasal cannula for 10 minutes prior to the procedure [14]. Patients with liver, kidney, and heart failure, as well as those allergic to magnesium (Mg) and propofol, were excluded from the study. Patients who were administered Mg were assigned to the M group, while those who did not were assigned to the Control (C) group.

Sedoanalgesia is routinely administered to patients undergoing EBUS in our clinic. Some clinicians administer inhaled or intravenous Mg for bronchodilation prior to the procedure. These patients were given 10 ml of 15% MgSO4 (1500 mg/10ml). Thirty minutes before the procedure, 3 ml (450 mg) of this was administered via inhalation for 10 minutes. The remaining 7 ml (1050 mg) was mixed with 100 ml serum physiologique and administered intravenously over 30 minutes. Patients who received different MgSO4 regimens or sedoanalgesia than described above were excluded from the study.

Electrocardiography (ECG), heart rate (HR), oxygen saturation measured by pulse oximeter (SPO2), noninvasive blood pressure (NIBP), IPI monitor, and bispectral index (BIS) are all used to monitor patients undergoing EBUS in the operating room. Data obtained from the capnograph monitor's nasal probe (end-tidal carbon dioxide (EtCO2), respiratory rate (SS) and the finger probe (SPO2 and HR) are used by the IPI monitor (Capnostream® 20p/Covidien; Medtronic, Dublin, Ireland) to calculate a score that indicates the respiratory condition of the patient. IPI values range from 1 to 10, with values between 7 and 10 reflecting acceptable respiratory parameters and values below 7 requiring attention (Table 1) [10,11].

**Table 1:** IPI scoring table IPI - Integrated Pulmonary Index

IPI	Patient's status
10	Normal
8-9	Within normal range
7	Close to normal range, requires attention
5-6	Requires attention and possible intervention
3-4	Requires intervention
1-2	Requires emergency intervention

At the start of EBUS, patients were given an intubation score (ranging from 3 to 12, with higher values indicating better intubation conditions), which assessed vocal cord mobility, cough reflex, and leg movement during bronchoscope insertion [15]. For sedation, propofol was administered intravenously to all patients. The dose of propofol was adjusted so that BIS values were between 60 and 80, the patient could tolerate the procedure, and spontaneous breathing was not suppressed. Patients were given 4-8 l/min O2 via nasal cannula during the procedure. After the procedure, the patients are transferred to the recovery unit and then to the ward after being monitored in the recovery unit.

Age, ASA classification, smoking status, Medical Research Council (MRC) dyspnea scale, intubation score, NIBP, mean arterial pressure (MAP), HR, SPO2, IPI score, BIS values during the procedure, duration of the procedure, presence of complications and the total amount of drugs used were recorded from patient files. Comparative analyses of propofol consumption, respiratory and hemodynamic parameters, IPI scores, intubation scores, and the presence of complications were conducted.

Statistics

SPSS version 18.0 (IBM Inc., Armonk, New York) was used to analyze data. The standard distribution measures of mean and standard deviation were used. In independent groups, comparisons were made using the t-test, Levene's test of variance equality, and Chi-square tests. The normal distribution was determined by using the Kolmogorov-Smirnov test, and the non-parametric Mann-Whitney U test was used to compare data. A p-value of <0.05 was regarded as statistically significant.

## Results

**Figure 1:**
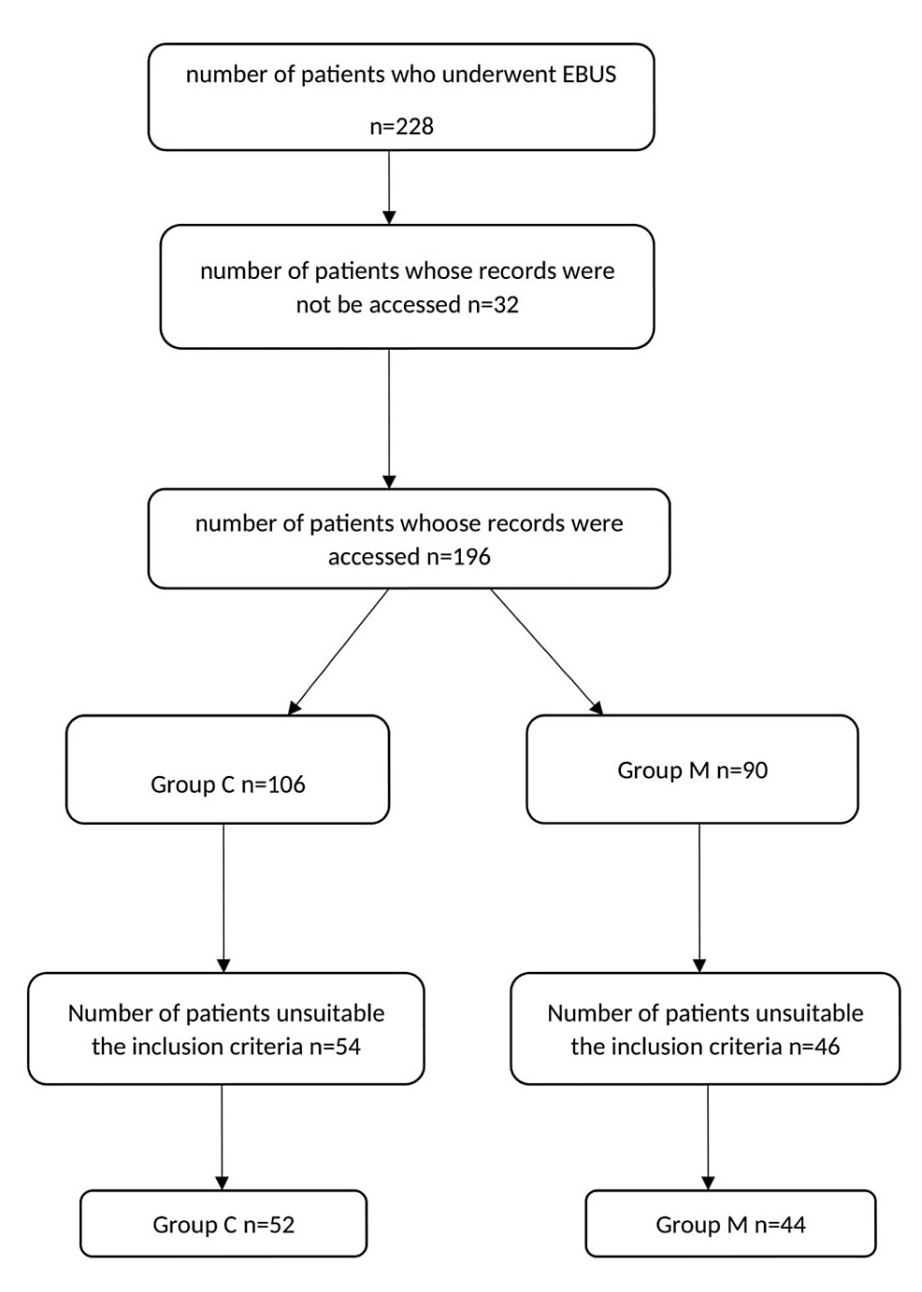
Flow chart

Our study included a total of 96 patients, 44 in group C and 52 in group M (Figure 1).

Age, gender, smoking status, ASA class, and MRC dyspnea scale scores of patients did not differ between the two groups. At the beginning of EBUS, there was no difference between the groups regarding SPO2, IPI, and BIS values. In both groups, the average procedure times were similar (p>0.05) (Table 2).

**Table 2:** Demographics and initial characteristics of patients ASA - American Society of Anesthesiology, MRC - Medical Research Council, IPI - Integrated Pulmonary Index, BIS - bispectral index

Variables	Control	Magnesium	p-value
Age (mean±SD)	58.84±10.96	52.32±8.55	>0.05
ASA II/III (n)	25/19	39/13	>0.05
Gender F/M (n)	14/30	16/36	>0.05
MRC dyspnea scale 0/1/2/3/ (n)	0/18/16/10	0/16/25/11	>0.05
Initial saturation (%) (mean±SD)	96.75±2.24	96.44±2.35	>0.05
Initial IPI (mean±SD)	8.7±1.37	8.5±1.68	>0.05
Initial BIS (mean±SD)	95.77±5.16	95.46±4.22	>0.05
Procedure length (min) (mean±SD)	20.43±7.7	19.48±6.2	>0.05

**Figure 2:**
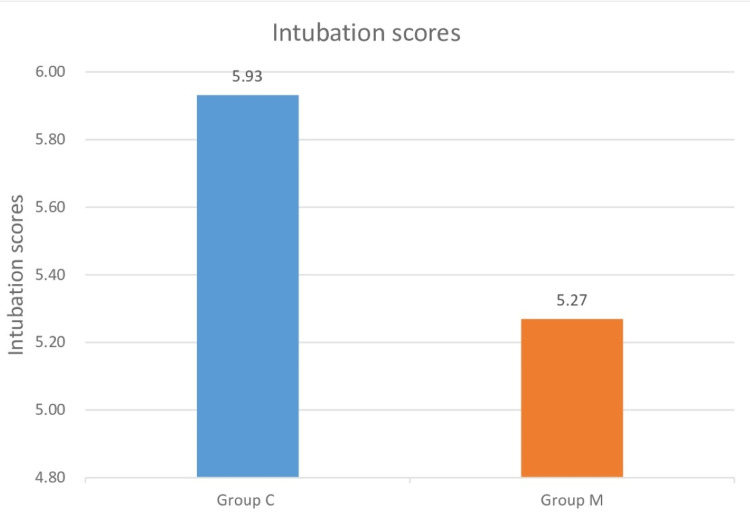
Comparison of intubation scores of group C and group M

During the EBUS procedure, the intubation score values obtained by observing vocal cord movement, cough reflex, and leg movement during the passage of the bronchoscope through the vocal cords were significantly higher than in group C (p<0.05). In other words, intubation conditions were significantly better in group M (Figure 2). 

**Figure 3:**
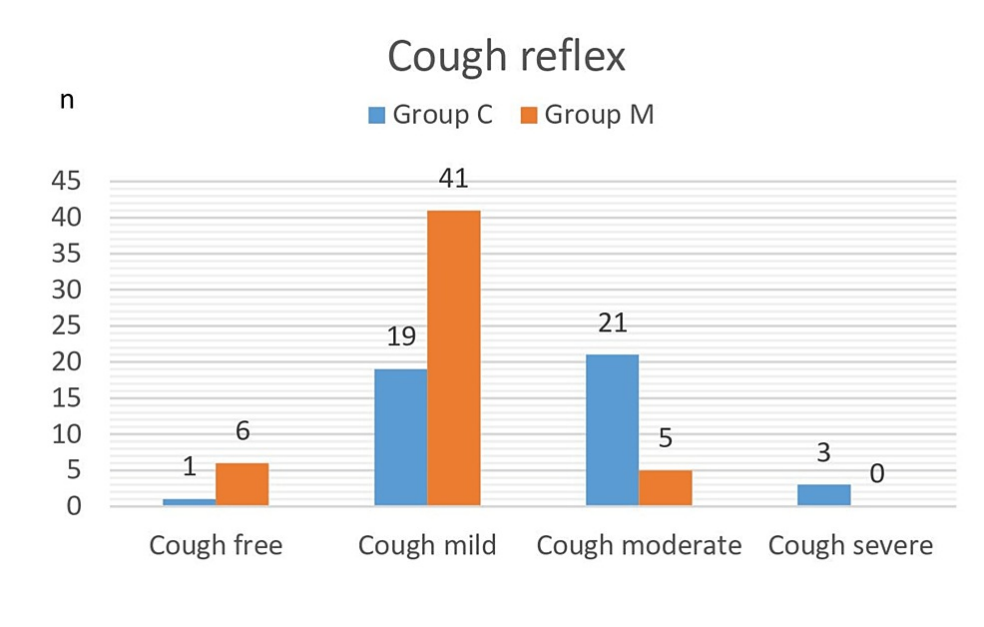
Comparison of cough reflexes between group C and group M

In the subgroup analysis of the intubation score, there was no difference between the groups in the assessments of vocal cord movement and leg movement (p>0.05). Still, there was a significantly lower cough reflex in group M (p=0.05). In group C, 45.5% of patients had a mild cough, while 54.5% had a moderate or severe cough. In group M, 90.4% of patients were cough-free or had a mild cough, while 9.6% had a moderate or severe cough. The intubation score was significantly lower for group M patients compared to group C patients in terms of cough reflex (p<0.05, Figure 3).

**Figure 4:**
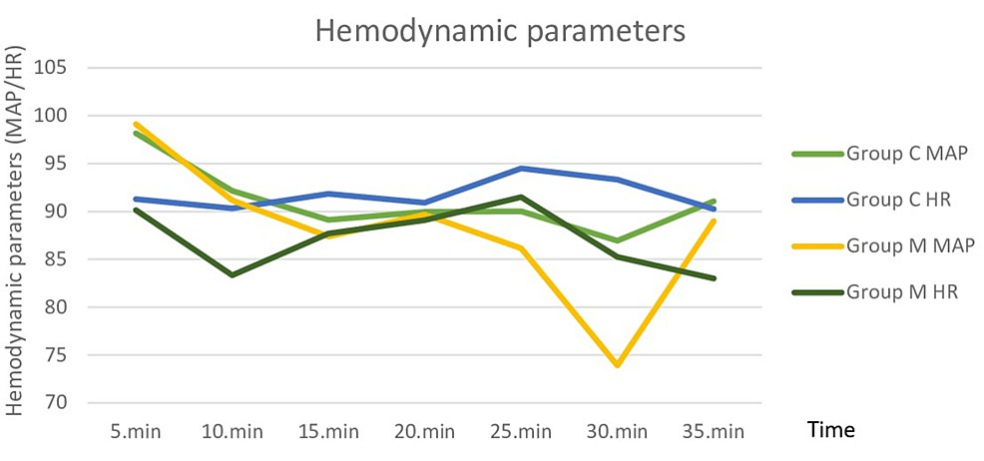
Comparison of the patients' mean arterial pressure and heart rate MAP - mean arterial pressure, HR - heart rate

MAP and HR data collected every five minutes during the EBUS procedure were similar between the groups (p<0.05, Figure 4).

**Figure 5:**
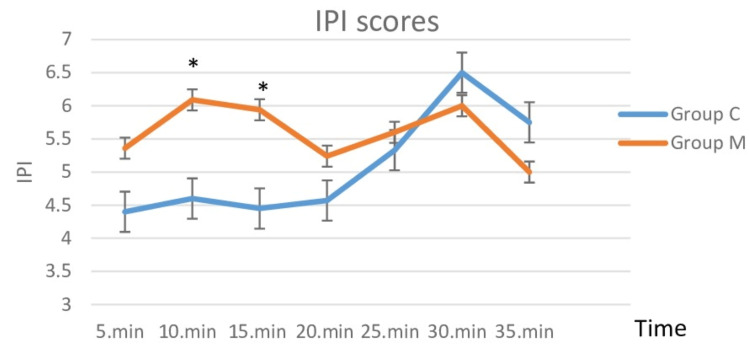
Patients' IPI scores IPI - Integrated Pulmonary Index * indicates statistical significance

The IPI scores in the M group were significantly higher in the tenth and fifteenth minutes compared to group C (Figure 5). No respiratory or hemodynamic complications preventing completion of the procedure were observed in either group.

**Figure 6:**
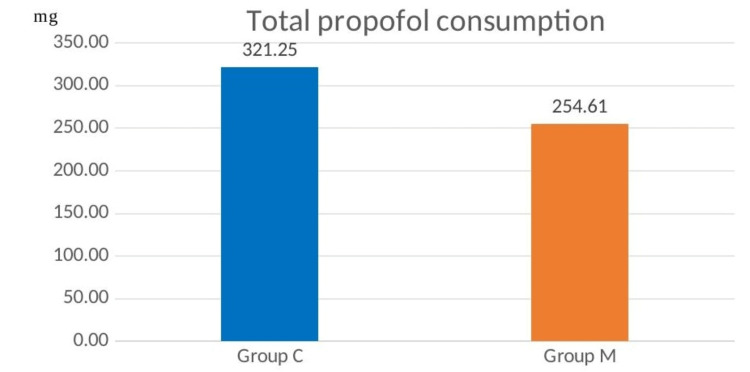
Comparison of total propofol consumption

For each patient, the total amount of propofol administered as mini boluses to ensure adequate sedation levels throughout the procedure was calculated. The average total propofol consumption in group M was lower when compared to group C (254.61±82.80 vs 321.25±90.04 mg, p<0.05, Figure 6).

**Figure 7:**
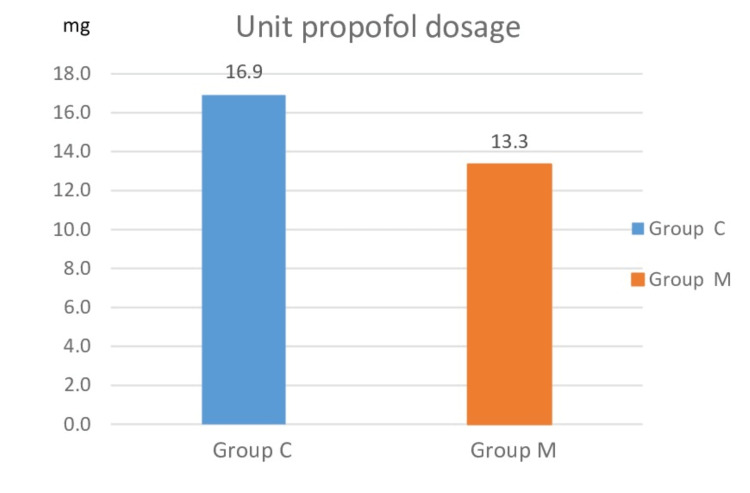
Comparison of unit propofol dosage between groups

The average amount of propofol administered in one minute was determined by dividing the total amount of propofol administered to each patient by the duration of the procedure (in minutes). The unit dose in group M was significantly lower than group C (13.43±3.43 vs. 16.9±4.82 mg, respectively, p<0.05, Figure 7).

## Discussion

In our study, the unit dose of propofol and the total amount of propofol consumption were found to be lower in patients who were administered IV or inhaled Mg when undergoing the EBUS-TBNA procedure under sedoanalgesia. In the Mg group, intubation score values were significantly lower than in the control group, indicating that intubation conditions were significantly improved. The cough reflex, which occurs upon passage of the bronchoscope through the vocal cord, was less prevalent in patients to whom Mg was administered. Furthermore, the IPI scores were higher in patients to whom Mg was administered, indicating that these patients had better respiratory function.

EBUS is a safe bronchoscopy technique with complication rates ranging from 0.08% to 6.8% [1]. These complications may be associated with either the EBUS procedure or sedation. Hypotension and hypoxia resulting from sedation. Bronchospasm, bleeding, pneumothorax, laryngospasm, and laryngeal edema may occur as a result of the procedure [1]. The use of various anesthetic agents and anesthesia levels have been reported for the EBUS procedure in literature. Ting-Yu-Lin [16] et al. reported that there was no difference in hypoxemia and recovery time between patients who received EBUS-TBNA sedation with dexmedetomidine or propofol. Aswanetmanee et al. [2] compared the effectiveness and safety of deep and moderate sedation used during the EBUS procedure and found that the length of the procedure, the cost, the patient's condition, and the doctor's level of expertise can all affect the outcome. All patients undergoing EBUS are routinely sedated to varying depths with propofol and BIS in our clinic.

During the EBUS procedure, it is important to closely monitor respiratory parameters. Pulse oximetry may be inadequate for real-time monitoring of hypoxia resulting from respiratory distress in patients undergoing follow-up care [17]. By tracking oxygenation with pulse oximetry and ventilation with capnography, we can specifically monitor hypercarbia and hypoxic respiratory failure. On the other hand, IPI is a monitoring technique that evaluates both oxygenation and ventilation by delivering noninvasive, dynamic, real-time, and continuous measurements. It reflects the respiratory state with high specificity and sensitivity, allowing for early identification of potential issues [12]. As it provides real-time monitoring of respiratory parameters, the IPI algorithm could help with decision-making during routine follow-up and recovery, according to a study conducted on patients who underwent electroconvulsive therapy [18]. It was suggested that the IPI monitor could be a useful measurement method for evaluating respiratory performance during propofol sedation in a study aimed at determining the predictive status of the IPI monitor for hypoxic pulmonary events in patients undergoing propofol sedation during the percutaneous endoscopic gastrostomy (PEG) procedure [10]. By monitoring our patients' respiratory status with IPI, we were able to detect hypoxia sooner and take preventative measures. When the IPI value dropped to 4 or lower, we performed maneuvers to stimulate the patient's respiration and decreased the drug dose, preventing hypoxia and its complications.

There are multiple mechanisms through which Mg can mediate bronchodilation. These include but are not limited to, attenuation of calcium-induced muscle contraction, suppression of cholinergic neuromuscular transmission, inhibition of inflammation, potentiation of beta-agonists, agonistic activity on adenylate cyclase, and relaxation of vascular smooth muscle mediated by prostaglandins. Moreover, Mg has anxiolytic and muscle-relaxing effects in cases of acute bronchoconstriction, in addition to its mild sedative effect [19]. In this way, the Mg group had a weaker cough reflex than the control group, and the Mg group had significantly higher IPI scores at 10 and 15 minutes than the control group. In patients undergoing propofol-sedation colonoscopies, Mg has been shown to significantly reduce the dose of propofol and improve respiratory and hemodynamic complications [20]. In the same study, it was observed that the sedation level was reached faster in the propofol and Mg group than in the group that did not receive Mg [20]. We found that the Mg group required less propofol than the control group during the EBUS procedure, despite our best efforts to maintain a BIS value of 60-80. This demonstrated that a reduced dose of propofol is sufficient to achieve the desired level of sedation when Mg is also administered at the same time. Sufentanil doses were found to be significantly lower in the group given Mg in addition to sufentanil, without a significant change in BIS values, in a study of patients who were sedated while undergoing mechanical ventilation (MV) treatment in the intensive care unit (ICU) [21]. Patients requiring MV support in the ICU who were administered Mg IV bolus and infusion were found to require less midazolam for sedation, fewer analgesics, and a shorter length of time on MV in another study [7]. In a study comparing patients who underwent abdominal surgery with and without IV Mg, Peinmandi et al. [22] found that patients who received IV Mg had significantly lower postoperative morphine consumption. As a calcium channel blocker, Mg amplifies the analgesic effects of morphine, and as an NMDA receptor antagonist, N-methyl has antinociceptive effects. Additionally, Mg demonstrates this effect by limiting NMDA receptor sensitivity in the dorsal root after inflammation and tissue damage in the periphery [19]. Patients in our study who were administered Mg had a lower analgesic requirement, leading us to believe that a smaller dose of propofol would suffice.

Our study has some limitations. The potential for bias is elevated due to the study's retrospective nature. The study's sample size was small because individual anesthesiologists' sedoanalgesia protocols vary. Another limitation of our study is that it was a single-center study. Better results can be expected from studies that use standardized protocols and prospective groups with similar characteristics.

## Conclusions

In the EBUS-TBNA procedure performed with sedoanalgesia, the use of magnesium reduced propofol consumption and provided better intubation conditions. Furthermore, the IPI scores were higher in patients to whom Mg was administered, indicating that these patients had better respiratory function. The use of less propofol causes less respiratory depression, so better respiratory parameters and fewer side effects such as apnea. In conclusion, our research demonstrated that improving respiratory parameters and significantly reducing the propofol dose during the EBUS procedure could be achieved by adding Mg to propofol sedation. In EBUS patients, the use of intravenous and inhaler Mg, in addition to sedation, provided better respiratory parameters using less propofol. The use of Mg gave us better patient outcomes and a more comfortable procedure. Further studies are needed in terms of the sedative and analgesic effects of Mg.
